# Minireview on Novel Anti-infectious Treatment Options and Optimized Drug Regimens for Sepsis

**DOI:** 10.3389/fmed.2021.640740

**Published:** 2021-04-15

**Authors:** Maya Hites

**Affiliations:** Clinic of Infectious Diseases, Cliniques Universitaires de Bruxelles (CUB)-Erasme Hospital, Brussels, Belgium

**Keywords:** antibiotics, multidrug resistant organisms, appropriate therapy, pharmacokinetics, gram-negative bacteria

## Abstract

Sepsis, a life-threatening organ dysfunction caused by a dysregulated response to infection is a major public health concern, as it is a leading cause of mortality and critical illness worldwide. Antibiotics are one of the cornerstones of the treatment of sepsis; administering appropriate antibiotics in a rapid fashion to obtain adequate drug concentrations at the site of the infection can improve survival of patients. Nevertheless, it is a challenge for clinicians to do so. Indeed, clinicians today are regularly confronted with infections due to very resistant pathogens, and standard dosage regimens of antibiotics often do not provide adequate antibiotic concentrations at the site of the infection. We provide a narrative minireview of different anti-infectious treatments currently available and suggestions on how to deliver optimized dosage regimens to septic patients. Particular emphasis will be made on newly available anti-infectious therapies.

## Introduction

Sepsis, a life-threatening organ dysfunction caused by a dysregulated response to infection (1), is a major public health concern, as it is a leading cause of critical illness and mortality worldwide. Indeed, data extrapolations from high-income countries suggest annual global estimates of sepsis cases and deaths in hospitals of over 50 million and over 5 million, respectively (2). Antibiotics are one of the cornerstones of the treatment of sepsis; administering appropriate antibiotics, defined as those that have *in-vitro* activity against the pathogen(s) responsible for the infection, in a rapid fashion to obtain adequate drug concentrations at the site of infection can improve patient survival (3–6).

Nevertheless, to give appropriate antibiotics to patients with sepsis is a challenge for the clinician because the risk of being confronted with an infection due to multidrug resistant (MDR) bacteria, particularly amongst Gram-negative bacteria, is increasing worldwide (7). To illustrate this point, we can look at the epidemiological situation in the European Union (EU) over the last 15 years concerning 2 important pathogens, *Escherichia coli (E.coli)* and *Klebsiella pneumoniae (K. pneumoniae)*. The estimated number of attributable deaths due to third generation cephalosporin (3GC) resistant *E. coli* was four times greater in 2015 than in 2007 (8). Resistance to 3GC is primarily caused by extended spectrum ß-lactamase (ESBL) enzymes; a matter of concern is that infections due to ESBL producing *E. coli* can either be community acquired or healthcare associated. The numbers of infections and deaths due to *K. pneumoniae* carbapenem-resistant strains also increased by over 6-fold from 2015 to 2018 (8). *K. pneumoniae* is currently the most frequent carbapenem-resistant Gram-negative bacteria causing infections (9–11). Indeed, *K. pneumonia* is 6–11 times more frequently responsible for carbapenem-resistant infections than *E. coli* (8, 12). The resistance to carbapenems, one of the last resort antibiotics, is often combined with resistance to other key antimicrobial groups, resulting in inappropriate initial therapy, but also suboptimal definitive therapy (8). Geographical distribution of resistance is highly heterogeneous, but the increasing rates of antimicrobial resistance is global (13).

To further complicate matters, it is also a challenge for clinicians to give adequate dosage regimens of antibiotics to patients with sepsis because standard dosage regimens often do not provide adequate antibiotic concentrations at the site of the infection (3). Standard drug regimens of antibiotics are determined from pharmacokinetic (PK) clinical studies in non-critically ill patients. However, when patients present severe infections, such as sepsis and septic shock, the PKs, or the concentration of antibiotics over time, particularly those that are hydrophilic (e.g., ß-lactams, aminoglycosides, and glycopeptides), are significantly altered, resulting in significant inter and intra-individual PK variability. These antibiotics have a distribution that is essentially extra-cellular, and their total body clearance (CL) is mainly dependent on renal mechanisms. Antibiotic concentrations may be lower than expected because of increased volume of distribution (Vd) due to fluid resuscitation, venous pooling, and hypoalbuminemia, but also due to increased drug CL because of high cardiac output and augmented renal clearance (ARC) (14, 15). Microvascular injury may also impair drug delivery to certain tissues (16). Low antibiotic concentrations may result in therapeutic failure, and/or facilitate emergence of resistance. Antibiotic concentrations may also be higher than expected, with risk of toxicity, because of decreased drug CL, particularly due to renal insufficiency. Toxic concentrations of glycopeptides and aminoglycosides are known to increase the risk of renal insufficiency (17), and accumulating data suggest that increased concentrations of ß-lactams can cause neurotoxicity (18). The PKs of antibiotics can be further altered by different renal replacement techniques and the use of extra-corporeal membrane oxygenation (ECMO).

It is in this light that clinicians have tried to optimize available antibiotic treatment regimens (by trying to attain the greatest efficacy, with the least toxicity) in the case of sepsis and septic shock, particularly in the case of infections due to MDR bacteria. Clinicians have also been anxiously waiting for new, effective antibiotics. The antibiotic pipeline has barely been trickling (19), yet several new antibiotics have finally made it to the market over the last couple of years, with indications to treat infections due to some of these problematic MDR Gram-negative pathogens. However, these antibiotics were not tested extensively in septic patients before getting European Medicinal Agency (EMA) and/or Federal Drug Administration (FDA) approval. In the light of these newly available therapeutic agents, we have performed a narrative minireview on their optimal antibiotic regimens in case of sepsis or septic shock. The antibiotics that will be reviewed here are ones that have received FDA and EMA marketing approval over the last 5 years for MDR Gram-negative infections: ceftolozane-tazobactam, cefiderocol, ceftazidime-avibactam, meropenem-vaborbactam, imipenem-relebactam, and eravacycline.

## Materials and Methods

This narrative review is based on a literature search performed in the Pubmed/MEDLINE database using combinations of pertinent keywords (“pharmacokinetics,” “ICU,” “sepsis,” “septic shock,” “MDR,” “critically ill,” “ceftolozone-tazobactam,” “cefiderocol,” “ceftazidime-avibactam,” “meropenem-vaborbactam,” “imipenem-relebactam,” and “eravacycline”). Retrieved papers were reviewed, and further searches were conducted using the reference lists. The review was then organized into 4 chapters: (1) theoretical antibiotic Pharmacodynamic (PD) targets and optimized drug regimens, (2) PKs of novel antibiotics in septic patients and optimized drug regimens, (3) perspectives for the future, and (4) conclusions.

## Theoretical Antibiotic Pharmacodynamic Targets and Optimized Drug Regimens

In order to optimize an antibiotic dosage regimen, the desired target concentrations have to be known. In bacterial infections, the target concentration will depend on the minimal inhibitory concentration (MIC) of the infecting pathogen being treated, and on the PK/PD index (or the relationship between the dose and the effect obtained) that best describes the efficacy of the antibiotic. Antibiotics can indeed be divided into three different groups based on PK/PD indexes to schematically describe their efficacy:

- **Time dependent antibiotics (e.g., ß-lactam antibiotics):** efficacy is greatest when the serum concentration of the unbound fraction of the antibiotic remains above the MIC for a minimum period of time (fT > MIC). To increase efficacy, the time of the antibiotic infusion can be increased, even to the point of administering the antibiotics in continuous infusion (as long as the stability of the antibiotic permits).
- **Concentration-dependent antibiotics (e.g., aminoglycosides):** efficacy is greatest when a certain ratio of the peak concentration to the MIC of the pathogen during one dosing interval is attained (Cmax/MIC). To increase efficacy, the antibiotic dose can be increased to attain higher peak concentrations.
- **Concentration-dependent antibiotics with time dependency (e.g., glycopeptides, tetracyclines):** efficacy is greatest when a certain ratio of the area under the curve of the serum concentration of the free fraction of the antibiotic over time to the MIC of the pathogen is attained (AUC/MIC). To increase efficacy, the antibiotic dose and infusion time can be increased (20).

All of the novel antibiotics are ß-lactams, with the exception of eravacycline. We will therefore only discuss how to theoretically optimize dosage regimens for ß-lactams and tetracyclines in sepsis and/or septic shock.

### ß-Lactam Antibiotics

Although there is no consensus concerning what specific PD target should be aimed for when administering ß-lactam antibiotics, *in-vitro* and clinical data suggest that a value of at least 100% fT > 1xMIC, but possibly even 100% fT > 4xMIC, may be needed to be effective (21).

When standard dosage regimens of antibiotics are administered, a great proportion of patients have insufficient serum concentrations during the first 24 h of treatment (14). In order to obtain adequate serum concentrations as soon as possible after antibiotic treatment initiation, a loading dose based on PK model simulations has been suggested (22). However, these findings still need to be validated in a prospective study.

Maintenance doses are given after loading doses; these need to be adapted to the CL of the antibiotic. However, if an increased loading dose is not given to the patient, then data suggests that standard dosage regimens could be given during the first 48 h of treatment, regardless of the renal function, and then adapted to the creatinine clearance based on 8–24 h urine collects (23). Indeed, initial standard drug regimens allow for better early PD target attainment in patients undergoing continuous renal replacement therapy (CRRT) (24). Furthermore, acute kidney injury (AKI) is frequent in patients admitted for severe infections, but can resolve rapidly after admission, as shown in a retrospective study evaluating data from a clinical database on 18,500 patients with predominantly pneumonia, intra-abdominal and skin-infections, where almost 1 in 5 patients presented AKI upon admission but was resolved within 48 h in over 50% of them (25).

To maintain serum concentrations above the MIC of the pathogen for the longest period of time, ß-lactam antibiotics can be administered as extended or continuous infusions. Although this improves PK/PD target attainment, it still remains to be proven that this mode of administration improves clinical outcomes (26). Indeed, extended or continuous infusions may require different PK/PD targets than intermittent infusions to get the same level of bacterial cell kill (27).

As clinicians aim to attain PD targets rapidly, the risk of reaching toxic concentrations increases. Nevertheless, there is currently no consensus concerning thresholds for ß-lactam toxicity; concentrations >10 times the MIC of the pathogen is the most frequently one currently used (22, 28).

Finally, to guide ß-lactam treatment, therapeutic drug monitoring (TDM) should be used in a routine fashion, as recommended in the recent position paper on antimicrobial TDM in critically ill patients, on behalf of different international scientific societies (28). Nevertheless, one of the challenges is that few centers have access to TDM of ß-lactams (29).

### Tetracyclines

Tetracyclines are not first choices to treat patients in sepsis or septic shock because only tigecycline is formulated for intravenous formulation, and early on during its' market-life, the FDA emitted a black box warning due to concerns about increased mortality associated with this antibiotic. The increased risk of death has since been attributed to therapeutic failure due to underdosing. Because the efficacy of this drug is best described by the PK/PD parameter, AUC _0−24_/MIC, to optimize PD target attainment, doses and frequency of administration can be increased. By increasing dosage regimens (i.e., 200 mg loading dose followed by 100 mg q12h, instead of 100 mg loading dose followed by 50 mg q12h), increased PD target attainment with better clinical outcomes than with smaller dosage regimens has been observed (30).

## Novel Antibiotics

### Ceftolozane-Tazobactam

This is a new antibiotic composed of a new cephalosporin associated with tazobactam, a well-known ß-lactamase inhibitor. It is active against MDR *Pseudomonas aeruginosa*, even those that are carbapenem-resistant, and ESBL producing Enterobacteriaceae. However, it is not active against carbapenem-resistant Enterobacteriaceae (CRE) (31). The drug is approved by the FDA and EMA for the treatment of intra-abdominal infections, complicated urinary tract infections, hospital acquired and ventilator-associated bacterial pneumonia (VAP). The antibiotic is also used by clinicians for off-label indications, particularly when faced with infections due to resistant pathogens with few or no other treatment options. The standard drug regimen is 1.5 g q8h, and 3 g q8h for VAP (Table 1) (37).

**Table 1:** PK/PD indexes and drug dosage regimens in adults of these novel antibiotics.

**Antibiotic**	**PK/PD index for efficacy**	**Standard drug dosage regimen**	**Drug regimens in septic or critically ill patients**
Ceftolozane-tazobactam (32)	%fT > MIC	Always to be administered in 1 h Creatinine clearance^*^: >50 mL/min: 1.5 g q8h 30–50 mL/min: 750 mg q8h. 15–29 mL/min: 375 mg q8h. End stage renal disease on hemodialysis: 750 mg, followed by 150 mg q8h	Always to be administered in 1 h Creatinine clearance^*^: >50 mL/min: 3 g q8h or. 1.5 g followed by 4.5 g CI 30–50 mL/min: 1.5 g q8h 15–29 mL/min: 750 mg q8h End stage renal disease on hemodialysis: 2.25 g followed by 450 mg q8h
Ceftazidime-avibactam (33)	%fT>MIC	Always to be administered in 2 h Creatinine clearance^*^: ≥ 50 mL/min: 2 g q8h 31–50 mL/min: 1.25 g q8h 16–30 mL/min: 0.75/0.1875 g q24h End stage renal disease or renal dialysis: 0.75/0.1875 g q24h	Always to be administered in 2 h Creatinine clearance: ≥ 50 mL/min: 2 g q8h 31–50 mL/min: 1.25 g q8h 16–30 mL/min: 0.75/0.1875 g q24h End stage renal disease or renal dialysis: 0.75/0.1875 g q24h
Cefiderocol (34)	%fT > MIC	Always to be administered in 3 h Creatinine clearance^*^: ≥ 120 mL/min (or ARC): 2 g q6h ≥ 60 ML/min: 2 g q8h ≥ 30 to < 60 mL/min: 1.5 g q8h ≥ 15 to < 30 mL/min: 1 g q8h End stage renal disease: 0.75 g q12h	Always to be administered in 3 h Creatinine clearance^*^: ≥ 120 mL/min (or ARC): 2 g q6h^**^
Meropenem-vaborbactam (35)	%fT > MIC	Always to be administered in 3 h Creatinine clearance^*^: ≥ 40 mL/min: 4 g q8h 20–39 mL/min: 2 g q8h 10–19 mL/min: 2 g q12h <10 mL/min: 1 g q12h	CVVH: Low efferent: 500 g q8h^**^ High efferent: 1 g q8h^**^
Imipenem-relebactam (36)	%fT > MIC	Infusion time: 30 min. Creatinine clearance^*^: ≥ 90–149 mL/min: 1,250 mg q6h ≥ 60–89 mL/min: 1,000 mg q6h ≥ 30–59 mL/min: 750 mg q6h ≥ 15–29 mL/min: 500 mg q6h End stage renal disease: 500 mg q6h	No data
Eravacycline (57)	AUC/MIC_0−24_	1 mg/kg q12h (even for obese patients) If taking CYP3A4 inducers: 1.5 mg/kg q 12h	No data

*PK/PD, Pharmacokinetic/Pharmacodynamic; %fT > MIC, percent of the time that the free fraction of the antibiotic remains above the minimal inhibitory concentration of the pathogen; AUC/MIC, area under the curve of the concentration of the free fraction of the antibiotic/MIC; CI, continuous infusion; ARC, augmented creatinine clearance; CVVH, continuous veno-venous hemofiltration*.

* *Based on the Cockcroft-Gault equation*.

** *Based on Monte Carlo simulations; dosage regimens not yet clinically validated*.

In a multicentric (22 hospitals), retrospective study in Italy from 2016 to 2018, 101 patients with serious and diverse infections due to *P. aeruginosa* (over 50% of strains were extensively resistant) were treated with this antibiotic with standard drug regimens. Clinical success (i.e., complete resolution of clinical signs/symptoms related to the infection and no microbiological evidence of infection) was observed in 83.5% of cases. However, lower success rates were observed in patients with sepsis or those receiving CRRT (37), possibly due to lack of PD target attainment in patients with sepsis. Indeed, in a population PK study of unbound ceftolozane and tazobactam in 12 critically ill patients without renal dysfunction, the probability of PD target attainment was ≥90% in patients receiving 1.5g q8h, but only for strains of *P. aeruginosa* with a MIC ≤2 mg/L. The 3g q8h regimen was needed to obtain a similar probability of PD target attainment for strains of *P. aeruginosa* with MICs of 4 mg/L. Finally, a loading dose followed by continuous infusion (1.5 g, then 4.5 g/24 h or 3 g, then 9 g/24 h) was needed to obtain optimal PD target attainment if MICs of *P. aeruginosa* were 8 and 16 mg/L, respectively (37). Another population PK model-guided evaluation of dosing in 6 patients undergoing continuous veno-venous hemodiafiltration (CVVHDF) showed that CL of ceftolozane-tazobactam is decreased in this situation. Nevertheless, in order to attain the PD target rapidly, a loading dose is necessary, followed by a reduced dosage regimen (Table 1). The optimal dosage regimen will depend on several factors, some being the specific CVVHDF settings, and the patient's residual renal function (38).

Finally, septic patients also sometimes benefit from ECMO. An *ex-vivo* model and then an *in-vivo* porcine model showed that ECMO has little influence on the PKs of ceftolozane/tazobactam, suggesting that no dose adaptation should be performed for these patients (39).

All of the proposed dosage regimens, particularly in the critically ill patients with sepsis still need to be validated in prospective clinical studies. No data is currently available concerning need for dosage adjustment in special patient populations such as the obese, the elderly, or patients with severe hepatic impairment.

### Ceftzidime-Avibactam

This is a combination of an existing cephalosporin, ceftazidime, and a new non-ß-lactam ß-lactamase inhibitor, avibactam which restores *in-vitro* activity of ß-lactams against Ambler class A [ESBLs and *Kl. pneumoniae* carbapenemases (KPC)], class C (AmpC), and certain enzymes from the class D (Oxa-48 carbapenemases). There is no activity against the Ambler class B enzymes (metallo-ß-lactamases), *Acinetobacter spp, Stenotrophomonas maltophilia*, or anaerobes. The antibiotic is registered by the FDA and the EMA to treat complicated intra-abdominal infections, complicated urinary tract infections, HAP, and VAP. The EMA has also approved its use for the treatment of infections due to Gram-negative bacteria for which few therapeutic options are available. The PK/PD index that best describes its efficacy is the %T> MIC. The standard dosage regimen is 2.5 g q8h for a patient with normal or augmented renal clearance, administered in 2 h. The doses must be decreased in patients with renal impairment (Table 1).

A population PK study on data from the five phase III trials was performed to validate these standard dosage regimens. The PK dataset for ceftazidime was made up of 9,155 observations from 1,975 subjects, and for avibactam, 13,735 observations from 2,249 subjects. A very wide range of clinical characteristics were captured, such as estimated creatinine clearances varying from 11 to 610 mL/min, and clinical presentations varying from the healthy volunteer to the patient with VAP. The covariates tested (that could have a possible significant effect on the PKs of ceftazidime and/or avibactam) were numerous, including disease status/indication of treatment, ARC, markers of systemic illness, severity of illness identified by the Acute Physiology and Chronic Health Evaluation version II (APACHE II), sex, age, obesity, body weight, race, and creatinine clearance. Creatinine clearance was the key covariate that predicted CL of both ceftazidime and avibactam. The covariates that affected the VD of both ceftazidime and avibactam were body weight, and mechanical ventilated patients when treated for nosocomial pneumonia, and treatment indication (40). Another PK study was carried out in 10 critically ill patients receiving ceftazidime-avibactam to once again evaluate adequacy of proposed standard dosage regimens (41). Both studies showed that standard dosage regimens resulted in a >90% probability of attaining the PD of 8 mg/L during >50% of the time for a relatively heterogenous patient population. However, this PD target is significantly lower than the suggested ß-Lactam targets of 100% fT> MIC or 100% fT> MICx4 in critically ill patients. Nevertheless, this standard dosage regimen of ceftazidime-avibactam has resulted in superior clinical success and survival than other treatment regimens to treat life-threatening infections by carbapenem-resistant pathogens (42, 43).

However, data on optimal dosage regimens in patients with septic shock, and those receiving CRRT are scarce. There are currently only 2 case reports with PK data in critically ill patients with infections due to MDR *Pseudomonas aeruginosa* (MIC: 8 mg/L). Both regimens (2.5 g q8h and 1.25 g q8h) attained the PD target of 100%fT > 4xMIC (44, 45).

### Cefiderocol

Cefiderocol is a catechol-type siderophore cephalosporin with very potent *in-vitro* activity against CRE and drug resistant non-fermenting Gram-negative bacilli. It uses the siderophore-iron complex pathway to penetrate Gram-negative membranes, like a Trojan horse. Once inside the bacteria, cefiderocol separates from the iron, and binds to penicillin-binding proteins to inhibit peptidoglycan synthesis. This antibiotic seems to have enhanced stability against hydrolysis by many ß-lactamases and carbapenemases. The antibiotic has been approved by the FDA and the EMA for Gram-Negative bacterial infections when other treatments might not work. The standard dose regimen is 2 g q8h in a 3 h infusion, but it needs to be reduced in case of renal insufficiency and increased in case of ARC (Table 1).

No PK data on cefiderocol is available in patients with sepsis, or patients in the ICU. However, in a phase III, double-blind, randomized trial for HAP, VAP, or health care associated pneumonia caused by Gram-negative pathogens, cefiderocol was non-inferior to meropenem concerning all-cause mortality at days 14 and 28 (46). On the other hand, in another phase III randomized (CREDIBLE-CR: NCT02714595), open-label study comparing cefiderocol to best available treatment (chosen by the investigator, with a combination of up to 3 drugs) for the treatment of severe infections caused by carbapenem-resistant Gram-negative pathogens, clinical cure rates were similar in both groups, however all-cause mortality was numerically higher in the cefiderocol arm on days 14, 29, and 49. The greatest imbalance with death was on day 49 in patients with APACHE II scores ≥ 16 and those with infections due to *Acinetobacter baumanni, Pseudomonas aerugi*nosa or *Stenotrophomonas maltophilia* (47).

Nevertheless, population-PK models were developed based on PK data obtained from phase I studies: plasma and urine concentrations from healthy individuals (*n* = 54) and plasma concentrations from individuals with varying renal function (*n* = 37). Monte Carlo simulations were then performed to determine optimal dosage regimens in patients with varying renal functions. Simulations showed that patients with ARC would benefit from an increased dosage regimen (i.e., 2 g q6h) (48). This proposed dosage regimen still needs to be validated in the clinical setting.

### Meropenem-Vaborbactam

Vaborbactam is a boron-based ß-lactamase inhibitor with activity against serine-ß-lactamases. It is a particularly potent inhibitor of KPC. When combined to meropenem, an existing carbapenem, it restores activity against KPC-producing carbapenem-resistant Enterobacteriaceae, both *in-vitro* and in preclinical models. The drug is approved for complicated urinary tract infections by the FDA, in addition to complicated intra-abdominal infections, and HAP, and VAP by the EMA. The standard drug regimen is 4 g q8 in 3 h infusions.

TANGO II is a randomized trial to compare the efficacy and safety of meropenem-vaborbactam to the best available therapy in adults with serious infections due to CRE. Seventy-seven patients were randomized, and 47 patients had confirmed CRE infections, but exclusion criteria were an APACHE score > 30, and an immediately life-threatening disease. Therefore, no patients with sepsis or septic shock were included in the study. Treatment with meropenem-vaborbactam for CRE infections was associated with improved outcome to best available treatment: increased cure, decreased mortality, and reduced nephrotoxicity (49). Nevertheless, no PK data is yet available in patients with severe infections.

An *ex-vivo* study has been performed to characterize the effects of continuous veno-venous hemofiltration therapy on the PKs of meropenem-vaborbactam. The study showed that there was little adsorption (<10%) of meropenem and vaborbactam by the extra-corporeal circuits. Furthermore, clearance of vaborbactam was 20–40% lower than the clearance of meropenem for an array of different settings and filters tested. Dosing according to currently optimized regimens for meropenem probably allows for adequate PD target attainment while ensuring adequate ß-lactamase inhibition during the entire dosing intervals: 500 mg/500 mg q8h for low effluent flow rates or 1 g/1 g q8h for higher effluent rates (50). Once again, these dosage regimens still need to be clinically validated.

### Imipenem-Relebactam

This is a combination of an existing carbapenem (imipenem-cilastatin) and a new non-ß-lactam ß-lactamase inhibitor, relabactam. It inhibits activities of certain ß-lactamases (Ambler Class A (e.g., KPC) and C (e.g., AmpC) cephalosporinases) but does not have activity against class B metallo-ß-lactamases, and class D carbapenemases. The addition of relebactam greatly improves the activity of imipenem against *Enterobacteriaceae* spp. (particularly those that are ESBL, KPC, and AmpC producing) and *Pseudomonas spp*. in function of the presence or absence of ß-lactamase enzymes. However, the activity of imipenem is not improved against *Acinetobacter baumannii*, and *Stenotrophomonas maltophilia* (51). The antibiotic is currently only approved for complicated urinary tract infections, including pyelonephritis. The dosage regimen is 500/250 mg q6h.

Only one trial has evaluated this antibiotic in ICU patients. In this phase III trial RESTORE-IMI 1, imipenem/relebactam was compared to imipenem and colistin in infections (HAP, VAP, complicated intra-abdominal infections, or complicated urinary tract infections) due to imipenem resistant bacteria. A favorable overall response was observed in 70% of patients; results were similar in both arms. However, over 50% of patients in the microbiological intention-to-treat arm presented a complicated urinary tract infection, and over two-thirds of patients had an APACHE score < 15 (52). Clinical and PK data from patients with sepsis or septic shock is clearly lacking.

### Eravacycline

This is the first fluorocycline antibiotic in the tetracycline class. It disrupts bacterial protein synthesis by binding to the 30S ribosomal unit. It has excellent *in-vitro* activity against Gram-negative bacteria (e.g., CRE, *A. baumannii* and *S. maltophilia*), but no activity against *Pseudomonas sp*. The PK/PD parameter that best describes its' efficacy is the AUC/MIC. The proposed current standard dosage regimen is 1 mg/kg q12h, to be increased to 1.5 mg/kg if patients are taking strong CYP3A4 inducers, with no drug dosage adjustment for renal or hepatic failure. Furthermore, this dosage regimen based on actual body weight appears to obtain high rates of clinical cure, without excess toxicity issues in obese patients. Indeed, cure rates were similar to those observed in obese patients treated with carbapenem containing regimens in a *post-hoc* analysis of pooled data from the Investigating Gram-negative Infections Treated with Eravacycline (IGNITE) 1 and IGNITE 4 phase 3 clinical trials comparing eravacycline to ertapenem and then to meropenem for complicated intra-abdominal infections. Drug discontinuation rates were no different between these two groups either (53).

This antibiotic has been approved both by the FDA and the EMA for complicated intra-abdominal infections, after showing non-inferiority to levofloxacin and ertapenem in the IGNITE 1 and 4 trials, where 80% of patients had APACHE II scores < 10. However, the antibiotic failed to show non-inferiority when used to treat complicated urinary tract infections when compared to levofloxacin in IGNITE 2, and ertapenem in IGNITE 3 because of poor urinary tract PKs (54). No other data is currently available concerning use of the antibiotic in bacteremia, in sepsis or septic shock.

## Perspectives for the Future

Clinicians need to remain vigilant concerning possible future therapeutic options because other antibiotics and anti-infectious agents, than those presented in this paper, are in the development pipeline. Table 2 provides a list of intravenous antibiotics active *in-vitro* against MDR Gram-negative bacteria currently in phase 1 to III trials (19). Because this paper reviews treatment options for patients with sepsis, only antibiotics with intravenous formulations have been presented.

**Table 2:** Intravenous antibiotics active *in-vitro* against gram-negative bacteria in phase I-III studies (19).

**Antibiotic**	**Compound**	**Clinical phases completed or on-going**
Benapenem	Carbapenem	Phase II trial completed in cUTIs: NCT04505683
SPR741 + ß-lactam	Polymyxin	Phase I trials completed: NCT03022175, NCT03376529
SPR206	Polymyxin	Phase I trial completed: NCT03792308
Apramycin	Aminoglycoside	Phase I trial completed: NCT04105205
Enmetazobactam (AAA 001) + cefepime	Clavulanic acid + 4th generation cephalosporin	Phase III trial on complicated urinary tract infections: completed: NCT03687255
Durlobactam (ETX2514) + sulbactam	Diazadicyclooctane-type ß-lactamase inhibitor + clavulanic acid type BLA inhibitor	Phase II completed for pyelonephritis and complicated urinary tract infections: NCT03445195 Phase III trial on-going: NCT03894046
Taniborbactam (VNRX-5133) + cefepime	Boronate + 4th generation cephalosporin	Phase III trial recruiting on complicated urinary tract infections: NCT03840148
Nacubactam (OP0595) + meropenem	Diazadicyclooctane-type ß-lactamase inhibitor + carbapenem	Phase I trials completed: NCT02134834, NCT02972255, NCT02975388, NCT03174795
Zidebactam + cefepime	Diazadicyclooctane-type ß-lactamase inhibitor + PBP2 + 4th generation cephalosporin	Phase I trials completed: NCT02532140, NCT02674347, NCT02707107, NCT02942810, NCT03630094
TP-6076	Tetracycline	Phase I active, not recruiting: NCT03691584

## Conclusions

New antibiotics active against MDR Gram-negative bacteria have finally made it to the market, providing the clinician with more arms to fight difficult-to-treat infections. Nevertheless, further evaluation of these novel antibiotics in the real-life setting is warranted. As a general rule, there is little data concerning the use of these novel antibiotics in patients with sepsis, particularly to treat infections due to MDR Gram-negative bacteria. Few post-marketing trials, including PK trials in special patient populations have been performed. Furthermore, case reports of rapid emergence of resistance to some of these antibiotics (e.g., ceftazidime-avibactam) have already been reported (55). Finally, these new antibiotics are significantly more costly than older antibiotics; they should only be administered to patients when no other effective therapeutic alternatives are available.

In order to use these new antibiotics in the most optimal fashion, more data (including PK data) on the efficacy of these antibiotics in critically ill patients, and those with sepsis or septic shock to treat MDR infections need to be obtained.

## Author Contributions

MH wrote the article on her own.

## Conflict of Interest

MH has received service fees from Pfizer and Gilead for participating in scientific and advisory board meetings in the domain of anti-fungal therapy.
